# Next Generation Sequencing Methods for Diagnosis of Epilepsy Syndromes

**DOI:** 10.3389/fgene.2018.00020

**Published:** 2018-02-07

**Authors:** Paul Dunn, Cassie L. Albury, Neven Maksemous, Miles C. Benton, Heidi G. Sutherland, Robert A. Smith, Larisa M. Haupt, Lyn R. Griffiths

**Affiliations:** Genomics Research Centre, School of Biomedical Sciences, Institute of Health and Biomedical Innovation, Queensland University of Technology, Brisbane, QLD, Australia

**Keywords:** next generation sequencing, epilepsy, gene panels, whole exome sequencing, whole genome sequencing, neurology, bioinformatics, d‘iagnostics

## Abstract

Epilepsy is a neurological disorder characterized by an increased predisposition for seizures. Although this definition suggests that it is a single disorder, epilepsy encompasses a group of disorders with diverse aetiologies and outcomes. A genetic basis for epilepsy syndromes has been postulated for several decades, with several mutations in specific genes identified that have increased our understanding of the genetic influence on epilepsies. With 70-80% of epilepsy cases identified to have a genetic cause, there are now hundreds of genes identified to be associated with epilepsy syndromes which can be analyzed using next generation sequencing (NGS) techniques such as targeted gene panels, whole exome sequencing (WES) and whole genome sequencing (WGS). For effective use of these methodologies, diagnostic laboratories and clinicians require information on the relevant workflows including analysis and sequencing depth to understand the specific clinical application and diagnostic capabilities of these gene sequencing techniques. As epilepsy is a complex disorder, the differences associated with each technique influence the ability to form a diagnosis along with an accurate detection of the genetic etiology of the disorder. In addition, for diagnostic testing, an important parameter is the cost-effectiveness and the specific diagnostic outcome of each technique. Here, we review these commonly used NGS techniques to determine their suitability for application to epilepsy genetic diagnostic testing.

## Key concepts:

1. Epilepsy is a syndrome which can present with a highly variable phenotype with genetic mutations thought to be the underlying cause in 70−80% of cases.
2. There is a large degree of phenotypic overlap between mutations in different genes associated with epilepsy syndromes and the utility of next generation sequencing technology can allow for the rapid identification of causative genetic mutations to influence a prognostic outcome and treatment options.
3. The key difference between current next generation sequencing techniques is the targeted enrichment step where gene panels focus on a limited number of genes; whole exome sequencing is focused on protein coding regions (~1−2% of the genome) and whole genome sequencing does not require targeted enrichment.
4. Gene panels target a set number of genes at a higher sequencing depth and lower cost when compared to whole exome and whole genome sequencing, however the number and specificity of genes included in the panel influences the success of diagnosis.
5. Whole genome sequencing can provide more even coverage of the genome and protein coding regions when compared to whole exome sequencing and gene panels, however there is a lower sequencing depth at a higher cost per sample.
6. Whole exome sequencing is associated with a high sequencing depth of the protein coding regions at a lower cost to whole genome sequencing.

## Introduction

Epilepsy is a neurological disorder characterized by an enduring predisposition to epileptic seizures. It has a prevalence of 4–8 per 1,000 and a lifetime risk of seizures in 3% in the general population (Fisher et al., 2005; Moller et al., 2015). Epilepsy is typically considered a multifactorial condition where seizures may only be one aspect of an underlying complex syndrome and the diagnosis encompasses considerable phenotypic heterogeneity. A genetic basis for some forms of epilepsy had been hypothesized for decades, and was confirmed via gene mapping in families and the identification of specific mutations associated with epilepsy syndromes in the 1990's (Annegers et al., 1982; Scheffer and Berkovic, 1997; Jallon et al., 2001; Myers and Mefford, 2015). It is now thought that 70–80% of epilepsy cases have a genetic cause, whilst the remaining 20–30% are due to acquired conditions such as stroke, brain trauma and tumors (Myers and Mefford, 2015).

The genetic etiology of epilepsy may be monogenic, resulting from single gene mutations (e.g., *SCN1A* mutations in Dravet syndrome). There are also polygenic forms involving mutations or variants in multiple genes are also thought to cause the disorder, although the genetic risk factors for these are less well understood (Scheffer and Berkovic, 1997; Moller et al., 2015). Currently, epilepsy genetics can be broadly characterized into two categories: (i) genes and loci associated with primary epilepsy; and (ii) genes associated with neurological disorders where epilepsy may be one of the symptoms (Poduri and Lowenstein, 2011). High throughput sequencing technologies have contributed to epilepsy gene discovery for both categories.

To date, extensive research has identified the genetic component of epilepsy syndromes with many different genetic aberrations now known to cause or contribute to the condition. Berkovic (2015) stated that genetic testing should be a common practice for clinical diagnosis of epilepsy syndromes. To accurately assess the utility of newer technologies for the diagnosis of epilepsy syndromes, it is important to be aware of the different types of genetic aberrations shown to be associated with epilepsy and seizure susceptibility and include a number of single gene mutations associated with epilepsy syndromes. A review by Wang et al. (2017) stratified 977 epilepsy-related genes into the following categories: 84 genes causing epilepsy as a core symptom; 73 neurological genes associated with brain gross development and epilepsy; 536 epilepsy-associated genes where epilepsy is a symptom of another neurological disorder; and 284 potential-epilepsy genes (Wang et al., 2017).

Chromosomal abnormalities, which can be detected via G-banded karyotyping, have also been linked with epilepsy. Ring chromosome 20 syndrome is a rare but well-known cause of epilepsy. Atkins et al. first described this condition in a 7 year old boy with behavioral issues, mental retardation and grand mal seizures (Atkins et al., 1972). Although not limited to these, other examples of chromosomal aberrations associated with epilepsy include Klinefelter Syndrome (47,XXY) (Elia et al., 1995; Tatum et al., 1998) and Pallister-Killian Syndrome (12p tetrasomy) (Pallister et al., 1977; Peltomaki et al., 1987).

Particular copy number variations (CNVs) have also been associated with epilepsy and other neurological disorders (Mullen et al., 2013; Mefford, 2015; Borlot et al., 2017). CNVs are classified as large >1 kb deletions or duplications of DNA which can be recognized as either a normal variation of the genome or to be pathogenic based on the location and number of genes within the variation (Mefford, 2014). Examples of CNVs associated with epilepsy include recurrent deletions at Xp22.31, 1q21.1, 15q11.2, 15q13.3, and 16p13.11 as well as duplications involving 1p36.33 and 22q11.2 which have all been previously identified as risk factors for genetic generalized epilepsy (Mefford, 2014; Addis et al., 2016).

Interestingly, other factors such as uniparental disomy (UPD) of chromosome 15 or genetic imprinting involving the region at 15q11-15q13 have also be associated with mild epilepsy in Angelman syndrome (Lalande et al., 1999; Valente et al., 2005). Like genomic imprinting, epigenetic factors have been implicated in epilepsy through the epileptogenic cascade including neuro-inflammatory responses, neuronal cell loss, mossy fiber sprouting, aberrant connectivity and gliosis with adenosine dysfunction (Roopra et al., 2012; Boison, 2016; Kobow and Blumcke, 2017). Epigenetic factors associated with epilepsy include DNA methylation (Kobow and Blumcke, 2012; Wang et al., 2016), histone modification and transcriptional regulation (Hwang et al., 2013; Jagirdar et al., 2015), and microRNAs (Henshall, 2014; Raoof et al., 2017). An example of how epilepsy is modified by transcriptional regulation is demonstrated by Repressor Element 1-Silencing Transcription factor (REST), a negative regulator of neuronal gene expression (Palm et al., 1998). Mutations in REST have been found to influence a number of genes including *HCN1* which is associated with temporal lobe epilepsy (McClelland et al., 2011).

Mitochondrial DNA (mtDNA) mutations have also been identified to cause epilepsy and other neurological diseases (Wallace et al., 1994). Examples were first seen by Shoffner et al. (1990) in 1990 where a mutation in *tRNA*^*Lys*^ was identified to cause myoclonic epilepsy with ragged red fibers (MERFF), and in 1992 where Tatuch et al. (1992) discovered a point mutation in *ATPase*^6^ causing Leigh syndrome if present in a high percentage of cells. Mosaic mutations in well-known epilepsy genes such as *SCN1A* and *SLC6A1* have also been identified to cause the epilepsy phenotype (Shi et al., 2012; Halvorsen et al., 2016). A study by Stosser et al. (2017) found a 3.5% overall frequency of mosaicism in 893 epilepsy probands across 9 different genes including *CDKL5, GABRA1, GABRG2, GRIN2B, KCNQ2, MECP2, PCDH19, SCN1A*, and *SCN2A*. Mosaicism is thought to be an underreported cause of genetic disorders, due to detection challenges, although there are numerous studies aimed at improving this using NGS technology (Stosser et al., 2017). Furthermore, mosaicism is not limited to single gene mutations or mtDNA, but can also be observed in chromosomal abnormalities and CNVs (Gajecka, 2016).

Next generation sequencing (NGS) is a relatively new technique now being applied to genetic testing. NGS has the potential to find causal mutations, including *de novo*, novel and familial mutations, associated with epilepsy syndromes and, due the variable phenotypic presentations of the disorder, vastly improve molecular diagnosis. First generation DNA sequencing with chain-terminating inhibitors invented by Sanger in 1977 (Sanger et al., 1977), led to many genetic discoveries and has been widely used for over 30 years in research and diagnostic laboratories. Although considered a major technological breakthrough, and still finding utility today for variant verification, the technique has limitations, in particular when examining large regions of the genome. More recently NGS has begun to replace Sanger sequencing due its ability to sequence large numbers of genes, the whole exome (protein-coding regions) or entire genome at once. Thus applications of NGS include targeted gene panels, whole exome sequencing (WES) and whole genome sequencing (WGS). Custom gene panel testing allows for screening of multiple potentially clinically relevant genes and for more flexibility in phenotype–genotype correlations than required when testing individual genes (Poduri et al., 2014). WES focusses on the protein coding regions in the genome, comprising approximately 1–2% of the genome, attributable to ~85% of disease related mutations (Choi et al., 2009). In contrast, WGS provides information on the entire genome (both coding and non-coding regions), providing additional information on mutations in regulatory regions, as well as copy number variations with higher efficiency than WES (Poduri et al., 2014; Stavropoulos et al., 2016). The ~99% of the genome not included as exome sequence also contains untranslated regions which may have a regulatory role (e.g., non-coding RNAs or transcription binding sites) along with potential protein coding sites yet to be annotated as genes (Chrystoja and Diamandis, 2014; Lohmann and Klein, 2014). The impact of variants found in non-coding regions are not currently well understood, however it is feasible that a single or a combination of variants could have a significant impact on the pathology of conditions such as epilepsy. This is most evident for non-coding variants that may influence expression levels or mRNA splicing, affecting protein abundance or isoforms.

Use of NGS technologies in research and diagnostic laboratories has given rise to the rapid identification of genes associated with epilepsy syndromes. Within this review, we will assess the suitability of these three NGS techniques with a particular focus on their application to aiding clinicians and laboratories for epilepsy diagnosis.

## Adaptation of the work-flow for epilepsy NGS

**Figure 1:**
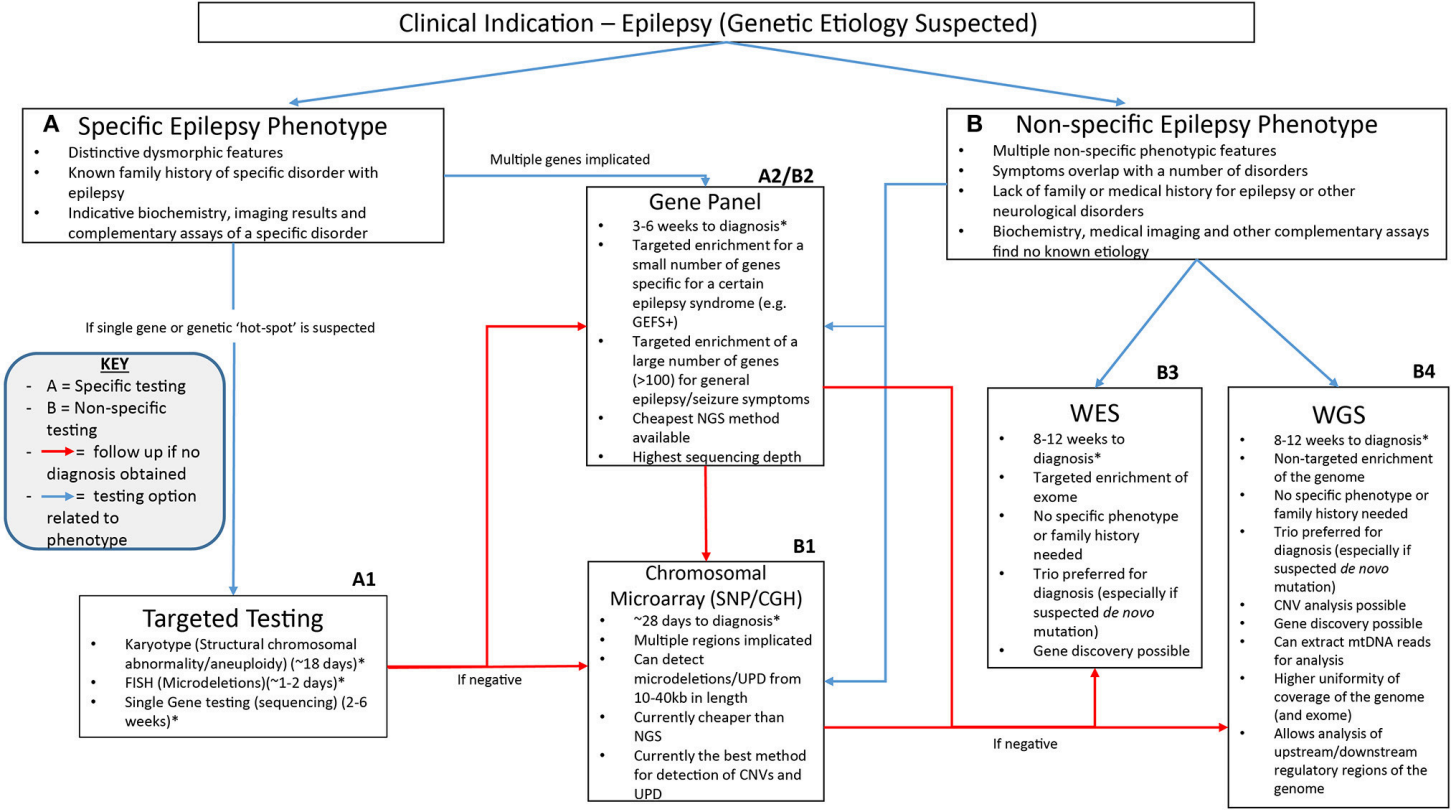
A suggested clinical workflow for identifying the genetic cause of epilepsy. This figure was adapted based off Xue et al. (2015). The use of genetic testing for epilepsy diagnosis needs to be determined based on how specific certain symptoms are. Karyotypes, single gene testing and FISH can be successfully utilized when a certain well-characterized disorder with epilepsy is considered. Gene panels can be used when specific phenotype—genotype correlation is proposed or for non-specific causes when a large number of genes can be included on the panel. Chromosomal microarray testing allows non-specific analysis of CNVs as well as uniparental disomy which may be associated with non-specific symptoms. Non-specific genetic testing is where WES and WGS can be best utilized as they can provide a non-phenotype derived approach to epilepsy diagnosis using little to no prior clinical information to provide a diagnosis. ^*^All times for diagnosis are conservative guides based off the turn around times stated from commercial genetic testing companies sourced from the Genetic Testing Registry https://www.ncbi.nlm.nih.gov/gtr/. The turn-around times for diagnosis may differ depending on the laboratory performing the test.

The NGS workflow consists of multiple steps including library preparation and enrichment, sequencing, base calling, alignment to the reference genome and variant annotation (Samorodnitsky et al., 2015a). Increased use of this technology has given rise to a range of techniques for library preparation, amplification and chemistries in order to prepare the DNA for sequencing. The role of NGS in epilepsy can be seen in Figure 1 which highlights the advantages of clinical genetic testing using NGS in epilepsy diagnosis including relevant advantages of each technique.

In all NGS approaches, DNA is fragmented prior to sequencing. This is performed in several ways and is dependent upon the specific commercial kit used for library preparation, and the sequencing platform. DNA can be sheared using high frequency soundwaves (sonication), via enzymatic digestion or transposase, or more recently using an approach where specific pools of amplicons are bound to the DNA fragments to amplify target regions to be sequenced in massively parallel PCRs (Chang and Li, 2013; Samorodnitsky et al., 2015a). The key differences between the different assays is summarized in Table 1 which includes the current commonly utilized fragmentation and hybridisation techniques utilized by the common commercial assays. Depending on the method used, a bias may be attributed due to differences in fragmentation, capture probes, and amplification efficiencies (Sims et al., 2014). This bias is an important consideration when deciding between techniques and commercial kits as each approach can differentially impact target sequencing, depth and uniformity of on-target sequencing, GC content on the target capture (for WES and gene panels), as well as the performance of single nucleotide variation (SNV) and copy number variation (CNV) detection (Samorodnitsky et al., 2015a).

**Table 1:** Key differences in commonly used commercial assays for NGS are highlighted based on the fragmentation technique and hybridisation methods.

**Company**	**Assay name**	**DNA fragmentation technique**	**Fragment type and hybridisation method**
ThermoFisher	AmpliSeq (Ion Torrent)	Primers bind to the genomic DNA creating known amplified target regions.	Primers amplify targeted regions resulting in overlapping amplicon panels
Agilent	HaloPlex	Transposase Digestion	Circular DNA probes align to capture regions of genomic DNA
Agilent	SureSelect	Sonication	Randomly sized DNA fragments are created and synthetic oligonucleotides then bind to regions of interest in solution
Illumina	Nextera	Restriction Enzyme Digestion	Evenly spaced, gapped probes bind to DNA. Paired-end sequencing is then used to fill the resultant gaps
Pacific Biosciences	Pacific Biosciences	Random shearing of DNA or amplification of specific sequences	Template fragments are ligated to hairpin adapters at each end, resulting in circular DNA with a constant ssDNA strand
Oxford Nanopore Technolgies	Oxford Nanopore Technologies	Optional fragmentation via Covaris g-TUBE™ using centrifugal force (NOTE: DNA shearing is not recommended when longer reads are required).	Template fragments are ligated to hairpin adapters at each end, resulting in circular DNA with a constant ssDNA strand
NanoString Technologies	nCounter Analysis System	Restriction enzyme digestion	Barcoded probes for targeted genes of interest bind to the DNA whilst another probe anchors the sequence target for sequencing

One key analytical difference between the three NGS methods is the number of variants identified. Approximately 3–4 million variants per individual are commonly identified through WGS and approximately 30,000–40,000 variants that differ to the reference genome per person are obtained by WES (Lohmann and Klein, 2014; Hegde et al., 2017). Although the increased content generated from WGS allows for a better chance at finding pathogenic variants, it also increases the incidence of actionable incidental findings. As such, it is very difficult to interpret variants outside protein coding regions with most laboratories initially focussing on performing WES or gene panels in order to reduce the risk of observing these actionable incidental findings (Belkadi et al., 2015). The prevalence of incidental findings and variants of unknown clinical significance (VoUS) is expected to continue to increase due to improved annotation of variants (Souzeau et al., 2016).

Due to the large amounts of data generated from NGS, even from small gene panels, bioinformatics pipelines are required to effectively process and evaluate the sequence information. These pipelines routinely consist of two main steps: (i) alignment of the sequence to a reference; and (ii) identification and variant calling (Elsensohn et al., 2017). As WES and WGS are broad genetic tests, targeted analysis of exome or genome data may be undertaken. e.g., several studies have successfully utilized targeted analysis of WES data in cardiomyopathy and focal epilepsy patients to identify likely causal mutations and pathogenic variants (Golbus et al., 2014; Perucca et al., 2017).

Despite these promising results, challenges remain in the development of effective bioinformatics pipelines due to the diversity of bioinformatics tools available and their approaches, giving rise to another layer of complexity in the use of NGS for diagnostic testing (Guo et al., 2015). *In silico* databases, such as those listed in Table 2 should be utilized when considering the minor allele frequency (MAF), regions of conservation and the pathogenic potential of identified variants (Carson et al., 2014; Leong et al., 2015; Richards et al., 2015). It is estimated that 90% or more of nonsynonymous variants detected in the genome are common with a frequency of ≥5% in the population and therefore unlikely to have a pathogenic effect (Foo et al., 2012). Further complexity occurs with limited information available in databases on implicated gene mutations, making the causal link to disease difficult. *In silico* prediction tools can be used to predict pathogenicity of novel single nucleotide variants (SNVs) (Leong et al., 2015). However, these tools have limitations and without further research or information, definitive diagnosis is often difficult (Wallis et al., 2013; Leong et al., 2015). With these acknowledged limitations *in silico* prediction tools, and methods such as Combined Annotation Dependent Depletion (CADD) scores, aim to address these shortcomings through utilization of an expandable framework of information with diverse annotations of genetic variation (Kircher et al., 2014). Additionally, current guidelines for variant classification indicate that use of *in silico* prediction tools alone should not be used to definitively classify a genetic variant as pathogenic or benign. Ultimately functional testing is often required to confirm the pathogenicity of a variant.

**Table 2:** List of databases and *in silico* tools (derived from Richards et al., 2015) which can be utilized for variant curation in NGS analysis.

Population Databases:	Exome Aggregation Consortium	http://exac.broadinstitute.org/
	Genome Aggregation Database	http://gnomad.broadinstitute.org/
	Exome Variant Server	http://evs.gs.washington.edu/EVS/
	1000 Genomes Project	http://www.internationalgenome.org/home
	dbSNP	https://www.ncbi.nlm.nih.gov/projects/SNP/
	dbVar	https://www.ncbi.nlm.nih.gov/dbvar
Disease Databases:	ClinVar	https://www.ncbi.nlm.nih.gov/clinvar/
	OMIM	https://www.omim.org/
	Human Gene Mutation Database	http://www.hgmd.cf.ac.uk/ac/index.php
Locus/disease/ethnic/other-specific database:	Human Genome Variation Database	http://www.hgvd.genome.med.kyoto-u.ac.jp/
	Leiden Open Variation Database	http://www.lovd.nl/3.0/home
	DECIPHER	https://decipher.sanger.ac.uk/
Sequence database:	NCBI Genome	https://www.ncbi.nlm.nih.gov/genome/
	RefSeqGene	https://www.ncbi.nlm.nih.gov/refseq/rsg/
	Locus Reference Genomic (LRG)	http://www.lrg-sequence.org/
	MitoMap	https://www.mitomap.org/MITOMAP
Prediction Tools:	PolyPhen	http://genetics.bwh.harvard.edu/pph2/
	SNPs&GO	http://snps.biofold.org/snps-and-go/snps-and-go.html
	SIFT	http://sift.jcvi.org/
	SNAP	http://www.bio-sof.com/snap
	CADD	http://cadd.gs.washington.edu/home
	PROVEAN	http://provean.jcvi.org/index.php
	MutationTaster	http://www.mutationtaster.org/
	dbNSFP	https://sites.google.com/site/jpopgen/dbNSFP

*The use of these resources can be paired with guidelines for determining pathogenicity of variants (e.g. American College of Medical Genetics guidelines)*.

## Sequencing depth and uniformity implications

For confident diagnosis of epilepsy syndromes, the depth of coverage (also known as sequencing depth) and uniformity of sequencing is an important consideration to determine which NGS technique is most suitable. Sequencing depth is directly related to accuracy of the sequence alignment and ability to call variants as it describes the number of times that a given nucleotide in the genome has been read in the experiment. The capacity of different sequencing platforms affecting the sequencing depth also relies on the level ofmultiplexing. Since most NGS platforms only sequence a specific number of bases in a single experiment, increasing the breadth of loci covered reduces the depth of the sequencing. For germline mutations, low to intermediate minimum sequencing depth is considered to be between 4X and 20X whilst high minimum sequencing depth is commonly thought to be 30X or greater (Telenti et al., 2016). High sequencing depth is needed to confidently sequence the genome with evidence that low sequencing depth (<10X) may result in the detection of only the wild type allele, even when a heterozygous variant is present (Lohmann and Klein, 2014; Telenti et al., 2016).

The use of NGS panels has to date met the need of diagnostic laboratories to deliver fast and accurate results of known disease genes for particular syndromes, incorporating phenotype based diagnostic approaches. Several studies of gene panels of numerous sizes found that a minimum sequencing depth of ~10–50X can be achieved depending on the size of the panel (LaDuca et al., 2017). To date, use of WES indicates approximately ~90% of the exome can be sequenced at a minimum coverage of ≥20X, with the average coverage depth for most platforms falling between 80 and 130X (Ankala et al., 2015; LaDuca et al., 2017). WGS at an average depth of 30X can achieve a minimum coverage of 10X over a breadth of 90% of the genome (Sims et al., 2014).

A common feature of NGS is the presence of uneven, or lack of, coverage when GC-rich and GC-poor regions of the genome are sequenced (Aird et al., 2011; Chen et al., 2013). This can also contribute to coverage bias and deviation from uniform distribution of reads across the genome or even error bias in the form of a deviation from expected mismatch, insertion, and deletion rates across the genome (Chen et al., 2013). This is not a new occurrence, with Sanger sequencing also known to demonstrate compression artifacts related to the base composition of GC-rich stretches (Aird et al., 2011). Bias can occur at many stages throughout the NGS workflow including during amplification and data analysis including the alignment of the sequencing data (Ross et al., 2013; Samorodnitsky et al., 2015b). Many platforms will use correction methods to counteract the impact of GC content, usually through measuring fragment counts and GC counts on a curve to estimate the conditional mean fragment count per GC value (Benjamini and Speed, 2012). In an effort to limit the GC bias, advances such as PCR-free WGS, may give more even coverage of the genome and be less sensitive to GC rich regions (Meienberg et al., 2016). Bias can also occur as a result of other elements of library preparation independent of GC content, such as amplification efficiency in PCR based gene panels or exome approaches, as well as probe binding efficiency in capture based library preparation.

The lack of uniformity of sequencing depth across WES and WGS contributes to the difficulty of detecting CNVs. As CNVs have a well-known genetic etiology in epilepsy syndromes and other neurological disorders, detection through NGS is an ongoing area of research (Zhao et al., 2013; Yamamoto et al., 2016; Wang et al., 2017). CNV analysis can be inferred using targeted gene panels, but is limited to the genes within the panel. However it may also be assessed in WES and WGS data through different computational strategies via analysis of short DNA fragment reads. In an extensive review by Zhao et al. (2013), five key computational strategies for CNV detection using NGS data were identified including: paired end mapping (PEM); split read (SR); read depth (RD); *de novo* assembly; and the combination of the other four methods. CNVs have been successfully analyzed in gene panel testing (Dimassi et al., 2015; Kerkhof et al., 2017), WES (Yamamoto et al., 2016; Wang et al., 2017), and WGS (Xi et al., 2016).

Currently, it has been widely established that NGS is able to detect germline mutations present in all cells, however somatic and mosaic mutations are difficult to identify. High sequence depth which is more commonly available in targeted gene panels aids in the detection of mosaic mutations with studies more commonly associated with somatic mutations relying on coverage >100X for accurate variant calling (Contini et al., 2015). Stosser et al. (2017) were able to detect mosaicism in epilepsy patients through gene panels and WES with the sequencing depth of mosaic variants ranging from 42X to 2574X. In addition, King et al. developed a method for detecting mosaicism (MrMosaic) (King et al., 2017) and determined the accuracy for detecting mosaicism in targeted NGS (gene panels and WES) and WGS increased with sequencing depth. An example of this is the detection of heteroplasmic variants in mtDNA sequences via WGS for epilepsy syndromes (Ding et al., 2015; Smith, 2016). The high copy number of mtDNA per cell, provides an abundance of mtDNA-derived reads in WGS data with the high sequencing depth allowing accurate assembly of the entire mitochondrial genome and detection of mosaicism (He et al., 2010; Li et al., 2010; Goto et al., 2011).

## Diagnostic capabilities of NGS for epilepsy

Identification of a genetic basis for epilepsy discovered through the use of NGS will drive changes and improvements in treatment options for patients. This has already been seen with the identification of *ALDH7A1* variants where treatment and management of epilepsy is reliant on daily supplements of pyridoxine (vitamin B6) (Hunt et al., 1954; Mills et al., 2006).

A study completed by Chambers et al. (2016) identified different commercial epilepsy panels that provided significant variability in the number of genes selected (between 70 and 465). This reflects two schools of thought: the first is a need to stringently select epilepsy genes to increase the probability of identifying causal variants whilst minimizing the detection of VoUS; the second suggests a higher number of genes included in the panel, should increase the likelihood of finding a causative mutation. A current list of commercial panels is summarized in Table 3 and illustrates differences in the number of panel genes, but also the number of genes that overlap between these panels and highlights the difficulties and inconsistencies associated with choosing commercial epilepsy panels. Only the Athena 232 gene panel encompasses all the genes from the other commercial panels (Ambry 101 gene panel and GeneDx 83 panel) and interestingly, these 2 smaller panels do not completely overlap.

**Table 3:** A summary of the comparison between 8 commercial epilepsy gene panels including the number of genes screened in each panel and the percentage of genes of similarity between the panels.

**Company**	**NUMBER OF OVERLAPPING GENES BETWEEN PANELS**															
	**Blueprints Genetics**		**Gene DX**		**Greenwood**		**Invitae**		**Ambry**		**NHS**		Courtagen^*^		**Athena**	
Blueprints Genetics			72	87%	88	61%	89	71%	91	90%	33	92%	136	29%	131	56%
GeneDX	72	37%			68	47%	72	58%	69	68%	27	75%	63	13%	83	36%
Greenwood	88	45%	68	82%			78	62%	80	79%	30	83%	116	25%	114	49%
Invitae	89	46%	72	87%	78	54%			87	86%	32	89%	81	17%	109	47%
Ambry	91	47%	69	83%	80	56%	87	70%			32	89%	98	21%	101	44%
NHS	33	17%	27	33%	30	21%	32	26%	32	32%			31	7%	33	14%
Courtagen^*^	136	70%	63	76%	116	81%	81	65%	98	97%	31	86%			172	74%
Athena	131	68%	**83**	**100%**	114	79%	109	87%	**101**	**100%**	33	92%	172	37%		

*In bold are the gene panels where every gene from the smaller sized panel is included in the larger size. All genes within the GeneDx and Ambry panels are included in the Athena 232 gene epilepsy panel, however only 68% of GeneDx epilepsy genes are included in the Ambry 101 panel and 82% in the GeneDx 83 epilepsy gene panel. All data from this Table is current as of May, 2017*.

* *Courtagen Life Sciences closed its diagnostic neurology testing business in July, 2017*.

In addition, the commercial epilepsy gene panels vary in the number of genes screened (see Table 3), contributing to differences in the diagnostic yield and clinical significance of the test performed. A study of the diagnostic yield of epilepsy gene panels in 2015 found the numbers varied between 35 and 265 genes investigated, with the diagnostic yields reported to be between 10 and 48.5 percent (Mercimek-Mahmutoglu et al., 2015). The largest epilepsy panel (265 gene) yielded a diagnostic rate of 48.5% from a cohort of 33 patients (Lemke et al., 2012; Mercimek-Mahmutoglu et al., 2015). Although this may seem to indicate that an increased diagnostic yield may be associated with a larger number of genes, another study utilizing a 67 gene epilepsy panel was identified to have a diagnostic yield of 47% (9/19) of patients (Della Mina et al., 2015). This result, albeit from a small sample size, indicates that a higher number of genes in a panel may not always be needed to obtain a diagnosis. Table 2 also highlights the variability between current gene panels for epilepsy syndromes, suggesting a panel incorporating all genes across the different panels would maximize diagnostic yield. A retrospective study by Mercimek-Mahmutoglu et al. (2015) reviewed the diagnostic yield associated with commercial epilepsy gene panels and found that a 38-gene panel may be sufficient to obtain a diagnosis in 93% of genetically diagnosed cases. Interestingly, this 38-gene panel would not contain *SCN8A*, which has been well documented to cause a severe epileptic encephalopathy. This highlights the limitations associated with gene panels, with mutations in a gene not included in the panel, including those identified to have an established association with a severe epilepsy phenotype, would not have been found (Hammer et al., 1993; O'Brien and Meisler, 2013; Mercimek-Mahmutoglu et al., 2015).

Although diagnostic rates of gene panels are comparable to WES (Wang et al., 2014), some studies have suggested that the utility of gene panel testing for germline mutations may be more useful as a first or second tier diagnostic tool (Figure 1) in conjunction with other clinical tests such as MRI, EEG, biochemical and hormone level blood tests and routine genetic testing such as CMA and karyotype analysis (Shashi et al., 2014; Aradhya et al., 2017; Mei et al., 2017). As such, in cases where gene panel testing does not provide a diagnosis, further testing through WES or WGS would be beneficial (Mei et al., 2017).

Currently, WES has a reported diagnostic rate of approximately 25% in patients without a prior diagnosis (Yang et al., 2013, 2014; Valencia et al., 2015). This is higher than other comparable genetic tests including chromosomal studies (karyotype analysis) (5–10%) and CMA (15–20%) (Yang et al., 2014). Interestingly, a study of Mendelian disorders also found that WES enabled diagnosis in disorders with a specific neurological finding in up to 31% of patients (Yang et al., 2013); with WGS identified to have a diagnostic yield in non-specific pediatric patients of 34% when compared with 8% via CMA. Other small studies focussing on the phenotypes of autism and intellectual disability have been able to achieve diagnostic rates of ~40 and ~60% respectively, through the use of WGS (Jiang et al., 2013; Gilissen et al., 2014; Stavropoulos et al., 2016).

With the advancement of NGS technologies, WES and WGS may be more useful in a clinical setting than gene panels to obtain a diagnosis or to identify causal mutations. The unbiased approach provided by WGS allows identification of the genetic cause of epilepsy syndromes when no diagnosis has been found and is increasingly recognized as a technology that could replace current commonly used techniques including karyotype and CMA (Meienberg et al., 2016). However, there is a need to overcome current concerns of the clinical application of this technology, including the increased risk of incidental findings as well as potential difficulties observed in variant curation (Dewey et al., 2014). Incidental findings, defined as genomic variants of potential actionable medical relevance unrelated to the condition (Krier and Green, 2013), can be minimized through targeted analysis of WES and WGS which may also speed up the rate of diagnosis.

## Cost effectiveness of NGS testing

In 2007, the cost to sequence an entire genome was ~$10 million US dollars. Currently, gene panels, WES and WGS can be performed at a fraction of the cost (Christensen et al., 2015). The threshold of the magic “$1000 genome” is becoming a reality, with gene panels and WES increasingly incorporated into the clinical setting (Lohmann and Klein, 2014; Christensen et al., 2015). Although there is currently very little information available on the cost-effectiveness of NGS technology, as the technology continues to advance and costs decrease, it seems inevitable that it becomes routine in the diagnostic setting (Deverka and Dreyfus, 2014; Christensen et al., 2015).

The ability of WES and WGS to identify causative genes in epilepsy syndromes will likely have cost implications for patients and their families. A measure of the clinical impact and cost-effectiveness of WES as a diagnostic test in a cohort identified 19 patients (n = 40) that had undergone at least 4 previous genetic tests prior to diagnosis by WES (Valencia et al., 2015). An unnecessary burden on patients and the healthcare system. WES or WGS will also identify incidental findings for clinically actionable genes (Richards et al., 2015). It has been postulated that these discoveries may lead to an improved quality of life at a slightly higher lifetime cost to the patient and healthcare system (Phillips et al., 2014). However, it is important to note that the cost-effectiveness for incidental findings that have been recommended by the American College of Medical Genetics (ACMG) have not been evaluated in economic studies and are therefore yet to be fully evaluated (Richards et al., 2015; Douglas et al., 2016).

The cost-effectiveness of NGS is critical for implementation in a clinical setting to appropriately improve diagnosis rates. Schofield et al. (2017) completed a cost-analysis comparison of gene panel and WES for use in neuromuscular disease (NMD) and determined the efficacy of gene panel testing increased diagnosis from a rate of 46% for traditional investigation, to 75% for the NMD panel and 79% for WES. The report also found the cost saving per diagnosis using the NMD panel was ~US$18,470 (AUD$23,390) when compared with a cost saving of ~US$10843 (AUD$13,732) for WES (Schofield et al., 2017), inclusive of the cost of testing saved if a custom gene panel or WES was used earlier in the diagnostic pipeline. As WGS is not routinely used in diagnostic testing it is difficult to determine the true cost effectiveness of this technique. The increased use of these sequencing technologies accompanied by the rapidly declining cost for completing the test will allow WGS to be a more accessible resource for diagnostic purposes in the near future.

Further reducing costs, the use of gene panels enables more libraries (genomic DNA, enriched exome or targeted genes) to be accommodated per sequencing run. DNA samples are barcoded enabling multiple patient samples and the data to be deconvolved to individuals after sequencing. Gene panels routinely accommodate up to 24 sample libraries onto a single sequencing run, reducing sequencing costs to approximately AUD$150 (US$120) per sample (Saudi Mendeliome, 2015). This cost also reflects the lower volume of genetic data sequenced per sample in the panels. In comparison, between 3 and 12 samples can be completed per WES sequencing run depending on the platform used (Saudi Mendeliome, 2015). The quantity of sequence data in gene panels also provides a more rapid clinical interpretation of variants. The cost of WGS is currently 2–3 times that of WES, primarily due to the much larger volume of DNA sequenced (>15 times the volume of WES) (Lacey et al., 2014). However, Belkadi et al. (2015) have postulated that, at current costs, a reduction of 60% to the cost of WGS would make it as affordable as WES.

Cost reductions will also be influenced by the development of new sequencing platforms such as Oxford Nanopore Technologies (ONT) sequencing (Feng et al., 2015) and single molecule, real time (SMRT) sequencing by Pacific Biosciences (PacBio) (Rhoads and Au, 2015). The development of these techniques have eventuated due to a paradigm shift in the read length, from short reads (e.g., ~200 bp in AmpliSeq Ion Torrent systems) to longer reads (between 8 and 12 kb up to 200 kb in length) (Goodwin et al., 2016). The advantage of using longer read lengths include shorter sequencing time, resolution of hard to sequence AT/GC regions, as well as, detection of large structural abnormalities including deletions, insertions, inversions, translocations and tandem/interspersed regions (Pareek et al., 2011; Nakano et al., 2017). Although there is great potential in the application of these newer technologies to the diagnostic setting, the higher error rate (~13% for PacBio and 12–20% for ONT) as opposed to ~1% for current NGS, limit their suitability in a clinical setting for epilepsy diagnosis (Rhoads and Au, 2015; Goodwin et al., 2016; Lu et al., 2016).

## Conclusions

The use of NGS technologies is the future of genetic diagnosis. As further research is completed on epilepsy along with the identification of the causal variants associated with the disease, NGS will become the most viable diagnostic option. Presently, gene panel testing is the favored choice for epilepsy genetic diagnosis due to the lower cost and higher coverage of the technology. However, as the price for WES and WGS continues to decrease, they will soon be fully integrated into the diagnostic setting, providing a wider range of diagnostic options for clinicians to utilize.

## Author contributions

PD, LH, and HS: conceived the review. PD: developed the concept and drafted the manuscript. PD, CA, NM, MB, HS, RS, LH, and LG contributed to the structure and design of the manuscript, revised the manuscript and approved the final version.

### Conflict of interest statement

The authors declare that the research was conducted in the absence of any commercial or financial relationships that could be construed as a potential conflict of interest.
